# Outdoor cycling competence and balance control in older adults – cross-sectional analysis of the BiPerAge trial

**DOI:** 10.1186/s12877-026-08491-7

**Published:** 2026-10-19

**Authors:** Jana Rogler, Sebastian Krumpoch, Ellen Freiberger, Noam Margalit, Roee Zehavi, Itshak Melzer, Sharon Barak, Amir Shapiro, Dikla Judchak Amir, Shira Levi, Omri Lubovsky, Robert Kob

**Affiliations:** 1 Department of Internal Medicine-Geriatrics, Institute for Biomedicine of Aging, Friedrich- Alexander-Universität Erlangen-Nürnberg, Nürnberg, Germany; 2 Department of Physical Therapy, Faculty of Health Sciences, Ben-Gurion University of the Negev, Beer-Sheva, Israel; 3 Nursing Department, Faculty of Health Sciences, Ariel University, Ariel, 40700 Israel; 4 Department of Mechanical Engineering, Faculty of Engineering, Ben-Gurion University of the Negev, Beer-Sheva, Israel; 5 Department of Orthopedic Surgery, Barzilai Medical Center, Ashkelon, Israel

**Keywords:** Outdoor cycling, Balance control, Perturbation, Older adults

## Abstract

**Background:**

Although cycling offers numerous health benefits, age-related functional decline can increase the risk of accidents. Identifying balance components linked to outdoor cycling competence could inform targeted assessment and training. We aimed to exploratorily investigate the association between outdoor cycling competence and static, dynamic, and reactive balance control in older adults.

**Methods:**

In this exploratory cross-sectional analysis of baseline data from a two-center randomized controlled trial, 116 community-dwelling adults aged ≥ 65 years with ≥ 3 errors on a standardized outdoor cycling course completed a balance test battery: Short Physical Performance Battery (SPPB), including gait speed, Modified Balance Error Scoring System (MBESS) and Four Square Step Test (FSST). Cycling-specific trunk balance was assessed with the Perturbation Stationary Bicycle Robotics (PerStBiRo) system, which delivers unannounced angular-tilt perturbations during stationary cycling. We derived mediolateral (ML) trunk sway during unperturbed cycling (UPC) and trunk time-to-peak angle (s) and trunk sway (°) during perturbed cycling (PC). Spearman correlations and multivariable models adjusted for age, sex and country were used to examine associations with the number of errors in the standardized outdoor cycling course.

**Results:**

Participants (mean age 74.0 ± 5.4 years; 53% women) made 13.1 ± 8.0 errors on the outdoor cycling course. In bivariate analyses, more outdoor cycling errors were weakly associated with more total errors on the MBESS (r_s_ = 0.213, *p* = 0.022), slower gait speed (r_s_ = -0.203, *p* = 0.029), greater ML trunk sway during UPC (r_s_ = 0.246, *p* = 0.009), shorter trunk time-to-peak angle (r_s_ = -0.289, *p* = 0.002) and lower trunk sway in PC (r_s_ = -0.295, *p* = 0.002). In multivariable analyses, longer FSST time (B = 1.28 [95% CI 0.39, 2.17], *p* = 0.005) and greater trunk sway during UPC (B = 2.68 [0.60, 4.77], *p* = 0.012) were independently associated with more cycling errors.

**Conclusions:**

This study shows weak associations between outdoor cycling competence and dynamic balance control during both UPC and FSST. Low trunk sway during UPC on the PerStBiRo is weakly associated with better outdoor cycling competence. These findings could be further evaluated in future studies aiming to improve outdoor cycling competence.

**Trial registration:**

This is a retrospective analysis of BiPerAge study data registered at clinicaltrials.gov: NCT06453473 (initial submission: 11/06/2024), link: https://clinicaltrials.gov/study/NCT06453473.

**Supplementary Information:**

The online version contains supplementary material available at https://doi.org/10.1186/s12877-026-08491-7.

## Background

Few modes of transportation offer as many health benefits across age groups as cycling. Prospective cohort evidence indicates that regular cycling correlates with reduced all-cause mortality risk [1]. Older cyclists may particularly benefit from maintaining independent mobility and improving physical and mental health [2, 3]. Cycling is a feasible activity that benefits multiple physiological systems, including the cardiovascular and metabolic systems, promotes neuroplasticity [4, 5] and is associated with better balance control during standing [6].

Balance control is a fundamental component of human motor behavior, essential for maintaining stability during standing and walking [7, 8]. It depends on continuous integration of visual, vestibular, and somatosensory information with motor outputs to keep the body’s center of mass within the base of support under both predictable and unexpected conditions [9]. Because the human body is a multi-segment system, any voluntary movement during activities of daily living perturbs posture [8, 9]. These involuntary “automatic” movements are called anticipatory postural adjustments or proactive balance control, and are smoothly incorporated into our voluntary movement to ensure accurate and harmonious motion [9]. Therefore, effective balance control involves the ability to maintain postures, transition between them, and to recover from sudden unexpected perturbations, triggering automatic reflex-like reactive balance responses [10, 11].

During outdoor cycling, effective balance control remains essential for cycling stability in both expected and unexpected situations. Age-related decline in motor and sensory function appears to increase the risk of cycling accidents as older adult cyclists (aged ≥ 65 years) accounted for 45% of cycling fatalities in the European Union in 2012, rising to 50% in 2023 [12]. This increase may be explained by a higher proportion of older persons seeking to stay active for longer as well as the growing popularity of heavy, high-speed pedelecs in this population [12–14]. Hung and Lim [15] define a pedelec (pedal electric cycle) as a power-assisted bicycle equipped with an electric motor that supports the rider during pedaling. Importantly, it was found that among older adults, the most serious injuries occurred in single bicycle falls and crashes without the involvement of other road users and that pedelec riders were affected more often than conventional riders [12, 16]. The most common causes for these accidents were road-surface irregularities (e.g., narrow bicycle tracks or poorly visible obstacles), incorrect fitting of the bicycle, and loss of balance leading to falls while conducting balance-demanding maneuvers [12, 16, 17]. For older adults, especially (dis-)mounting a bicycle carries an increased risk of accidents [12, 16, 17]. Cycling competence in older adults therefore seems to be based mainly on the cyclists’ perceptual-cognitive abilities and effective balance control [18, 19].

Controlling trunk movements was found to reflect effective balance control, even following unexpected balance loss, as it enhances proximal stability, and may therefore provide a stable base for reactive limb movements [20, 21]. Trunk angle velocity has been associated with successful balance recovery after standing perturbations, following a trip during overground walking, and was highly involved in controlling reactive fixed base of support strategies [20, 22]. Smaller trunk movements (e.g., flexion and side flexion angles) at reactive foot contact following unexpected anterior-posterior and mediolateral (ML) balance perturbations in walking and standing are a strong predictor of successful balance recovery [20, 21, 23]. However, advancing age appears to be associated with greater trunk stiffness in older adults and other balance strategies during cycling may come into play, such as steering and knee movements [24, 25]. It remains unclear whether effective balance control in tasks where trunk control may be relevant is associated with older adults’ cycling competence.

To evaluate balance control in older adults, a comprehensive assessment of its components is required [7]. The most common outcome measures used to test balance and mobility in clinical practice are currently performance-based (e.g. Short Physical Performance Battery (SPPB), Four Square Step Test (FSST), Timed Up and Go). Static postural control likely contributes to a wide range of activities and evaluating it under progressively more challenging conditions (e.g., sensory manipulation, single-leg stance) may provide information about trunk-related balance abilities [26–28]. Since loss of balance during walking has been shown to be the leading cause of falls among older adults living in the community, it is further necessary to examine postural control in motion [11, 29]. That includes walking as a dynamic stability measure and reactive balance resulting from an unexpected externally provoked balance loss, known as perturbation [11, 29–32]. To provide a task-specific assessment of trunk reactive response during cycling we used the Perturbation Stationary Bicycle Robotics (PerStBiRo) system. This recently developed instrument enables quantification of trunk dynamic and reactive balance while cycling on a stationary bicycle mounted onto a moving platform [33]. Early studies demonstrated associations between bicycle perturbation-based balance training (PBBT) and performance in non-cycling static, dynamic and reactive balance tasks during standing and walking [34, 35]. Yet, it remains unclear which specific components of balance control may be associated with outdoor cycling competence in older adults. The current study incorporates objective quantitative measures of static, dynamic and reactive balance during standing, walking and cycling on the PerStBiRo system to assess associations with real-world cycling competence in older adults. This may provide a more in-depth understanding of the movement patterns of older cyclists and may inform targeted assessment and training of cycling-specific balance.

The aim of this explorative study was to investigate the association between outdoor cycling competence in older adults, operationalized as the number of errors on a standardized cycling course [18, 36] and different components of balance control, i.e., static, dynamic and reactive balance control. Subsequently, a multivariable analysis was conducted to estimate the independent relationship between errors in the cycling course and each balance instrument.

## Methods

### Study design

The aim of this study is examined by supplementary analyses of an ongoing prospective randomized controlled trial study, the BiPerAge study, and focuses on an exploratory cross-sectional analysis of the baseline data. The BiPerAge study is a parallel, single-blind randomized controlled trial investigating possible transfer effects of training on the PerStBiRo system on older adults’ cycling competence at two study centers: the Department of Physical Therapy at the Ben-Gurion University of the Negev, Beer-Sheva and the Institute for Biomedicine of Aging at the Friedrich-Alexander-Universität Erlangen-Nürnberg. All procedures were performed in accordance with the 1964 Helsinki declaration and its later amendments. Ethics approval was obtained by committees of the Ben-Gurion University of the Negev, Israel (#01-3321 Barzilai medical center, January 31, 2022) and Friedrich-Alexander-Universität Erlangen-Nürnberg, Germany, (#21-494-S, February 10th, 2022). All participants provided written informed consent.

The baseline assessments (evaluation of participants’ characteristics and physical function) were conducted at the respective study centers by trained research assistants. Cycling competence was assessed on a standardized outdoor cycling course within two weeks before baseline assessment, and the perturbation test on the PerStBiRo system was conducted within two weeks after completion of the baseline assessment. In the BiPerAge study, 116 participants were allocated to one of the two intervention groups with a ratio of 1:1 by block-randomization with randomly selected block sizes of 2, 4 or 6 stratified by study center. Group allocation was performed centrally by the leader of the intervention group, who is not involved in recruiting and scoring assessments using a list only showing the current allocation.

### Participants

Participants were recruited via advertisements in the local media (e.g., television, newspaper), flyers, and personal contacts using an in-house database from March 2025 until April 2026. Community-dwelling adults aged ≥ 65 years with objectively measured unsteadiness while cycling on a standardized bicycle (≥ 3 errors in cycling course) were enrolled. Participants that were not able to perform the cycling course at baseline could not participate in the intervention. Participants had to be able to travel to and from the cycling course and the assessments independently. Older adults were excluded if they had a severe acute or chronic illness that impaired their health and prevented them from safely participating in the study (e.g., ischemic heart disease that restricts exercise, uncontrolled blood pressure), scored ≤ 24 points in Mini-Mental State Examination [37] or weighed > 120 kg (exceeds stationary bicycle weight limits).

### Outcome measures

#### Outdoor cycling competence examination

Cycling competence was inversely operationalized by the errors on a standardized bicycle in an outdoor cycling course for older adults as main outcome [18, 36]. In the BiPerAge trial, the stations were partially adapted compared to the original design and contained 6 tasks requiring good balance and cycling skills relevant to everyday traffic situations: slalom, slow cycling, dismounting and mounting the bicycle to both left and right side, cycling through a narrow alley, turning left and cycling a figure eight (see Additional file 1). Compared to the SiFAr trial [18], the time for slow cycling through a corridor was extended from 5 to at least 10 s, and precise braking was replaced by cycling a figure eight. Before the measurement, participants walked through the cycling course together with a researcher, who instructed the upcoming tasks. The instructions were given by the same researcher (one in Israel and one in Germany), who also evaluated the cycling course afterwards. The researcher did not conduct any intervention training sessions and was blinded to group assignment. After performing a test ride, cycling errors were documented during a second ride by using a standardized error protocol (see Additional file 1). A total number of 0 to 57 errors was possible. In addition, the second ride was recorded by two cameras to compare the error protocol against the video material afterwards. At least three trained study staff members were present at each test session for reliable detection of errors and providing safe conditions. Wearing a helmet was mandatory. To reduce potential ceiling effects, the SiFAr trial [18] served as a benchmark, and only individuals with three or more errors at baseline were included in the BiPerAge trial. Consistency in the implementation and scoring of the cycling course was ensured through a face-to-face meeting between researchers from both centers prior to the start of the study and through monthly meetings.

#### Static balance examination

##### Short Physical Performance Battery

The SPPB assesses the lower extremity function of older adults. It includes three components: standing static balance, a timed 4-meter comfortable gait speed test, and five repeated chair rises [38]. Sub-scores were summed to an overall score (0–12 points).

For the static balance part, three positions with open eyes were performed in the following order: side-by-side, semi-tandem, and full tandem. Each test was timed with a stopwatch and continued until the participants moved their feet, grasped the examiner for support, or 10 s had elapsed. Any time less than 10 s was rated in accordance with the scoring sheet (0–1 point for side-by-side and semi-tandem, 0–2 points for full tandem). Three blinded raters conducted the SPPB (one in Israel, two in Germany). A systematic review shows that the SPPB demonstrates good to excellent test–retest reliability in older adults, with intraclass correlation coefficients (ICCs) ranging from 0.82 to 0.92, indicating high stability over repeated assessments [39].

##### Modified Balance Error Scoring System

The Modified Balance Error Scoring System (MBESS) has so far been carried out mainly with young, athletic adults to assess postural stability after concussion [40]. It can be used as a more challenging tool to assess static balance in older adults [41]. Each of the three tasks, side-by-side, single-leg and tandem stance, was performed with eyes closed, hands positioned on the hips and with socks on. Participants were instructed to hold each stance for 20 s and to try to regain it as quickly as possible when moved out of the position. For the single-leg condition, the participants were encouraged to stand on their non-dominant leg. Opening the eyes, stepping, stumbling or falling out of position and/or grasping to regain stability was documented as an error. The total error score varied between 0 and 30 points representing the deviations from the proper stance (up to 10 per position). Participants unable to maintain the testing stance at all or for at least five seconds were assigned the maximum number of errors for this task. The MBESS has moderate to good test–retest reliability (ICC = 0.60–0.88) across a variety of adult populations [42] although reliability estimates in community-dwelling older adults are less extensively studied.

#### Dynamic balance examination

##### Gait speed

To assess dynamic stability, the comfortable 4-meter walk in the SPPB was performed [30, 43]. The test was assessed using a marked walking course. The time was recorded with a handheld stopwatch and the fastest of two consecutive trials was used for scoring purposes and to compute gait speed. Participants were instructed to walk at their usual speed. The comfortable 4-meter walk test, a component of the SPPB, has demonstrated excellent test–retest reliability in older adults (ICC = 0.90–0.98) [44].

##### Four Square Step Test

The FSST is a validated tool to assess dynamic stability, stepping agility and mobility in older adults [45, 46]. The test required two sticks lying on the ground to form a cross and four squares. Starting in the bottom left square participants stepped into each square with both feet as quickly as possible, without touching the sticks, clockwise and counterclockwise to complete one trial. This required them to take forward, backward, and lateral steps to the right and left. If the participants felt unsure, they were allowed to turn their bodies in the direction of the next step. After a successful training trial, two FSSTs were completed with the faster time taken as the score. A trial had to be repeated if the participants failed to complete the sequence successfully or made contact with the sticks. The FSST is a valid and reliable measure of dynamic balance and mobility in older adults (ICC ≈ 0.81–0.99) [46].

#### Dynamic and reactive cycling-specific balance examination

##### Perturbation test

**Fig. 1:**
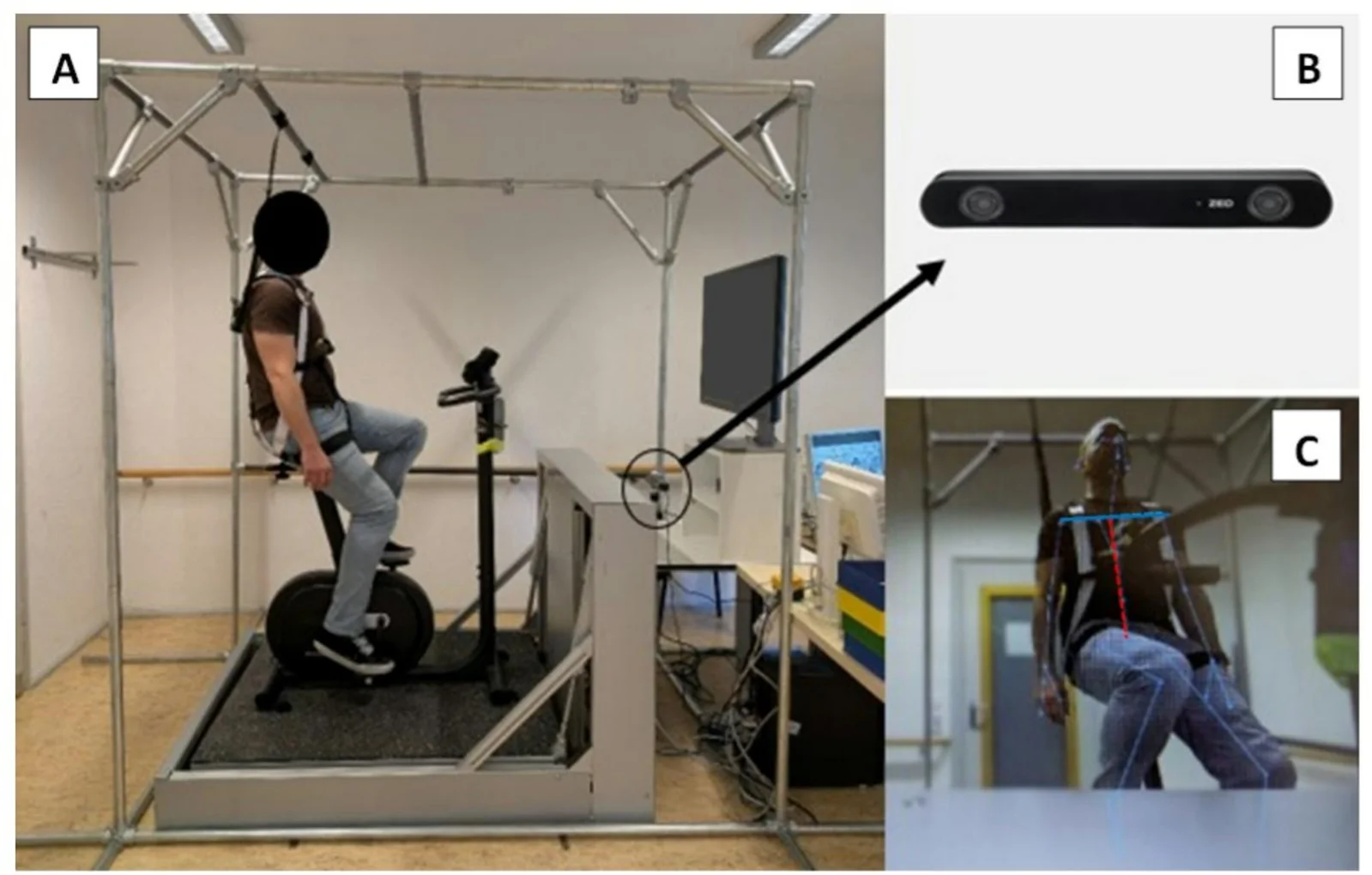
PerStBiRo system and motion capture. **A** Technical structure of the PerStBiRo system with researcher cycling hands-free and wearing a safety harness. **B** Stereolabs ZED 2i camera as motion capture system. **C** The motion capture system determined upper body joints (shoulder line and trunk line angle visible in blue and red, respectively) to detect dynamic sway and reactive balance responses

The PerStBiRo system was developed at the Ben-Gurion University of the Negev to assess and train cycling-specific trunk dynamic and reactive balance and consists of a stationary bicycle mounted onto a moving platform (Fig. 1A) [33]. The system provoked unannounced, programmed external perturbations in four different directions (right, left, forward, backward) with individualized angles (1°–10°) and velocities (10–500 mm/s). However, only ML perturbations but not backward and forward perturbations were analyzed in this study. Shoulder and trunk movements were recorded by the motion capture system Stereolabs ZED 2i camera (Stereolabs Inc., San Francisco, CA, USA) (Fig. 1B and C). All participants wore a loose safety harness that could arrest falling of the PerStBiRo system. The feasibility of the PerStBiRo system has been described in previous studies [34, 35] and more information on the setup and technical data has been published [33].

The perturbation test served to determine dynamic and reactive trunk control. During the whole session, participants were encouraged to pedal hands-free to maximize the balance challenge [9]. The participants were instructed as follows: “Try to compensate for the unannounced perturbation tilts with your upper body while continuously pedaling at 48–52 revolutions per minute. If you feel very unsteady or unsafe, you can hold onto the handlebars and we will stop the program.” The perturbation test began with a 3-minute warm-up during which the motion capture system detected the neutral position of the participants’ shoulder and trunk angles (calibration phase). This procedure minimized measurement errors and automatically customizes the PerStBiRo system to each participant’s sway during voluntary pedaling. It consisted of a 3-minute period of pedaling during which trunk and shoulder angles were recorded (i.e., trunk sway). These data were used to calculate the participant’s natural trunk position (zero point) and baseline trunk sway amplitude, accounting for individual posture and movement variability during pedaling. The resulting personalized thresholds enable the system to distinguish perturbation-induced balance reactions from normal pedaling movements. The zero point indicates the angle to which the PerStBiRo system returns within 3 s after a perturbation. In the main part, the participants performed a step protocol lasting up to 10 min at continuous resistance, during which both the perturbation angle and velocity increased gradually. In total, there were 43 perturbations during the main part, approximately four perturbations per minute, in random timing and direction (see Additional file 2). If the participants reached a response threshold value, specified in a previous study [34], within three seconds after the start of a perturbation, the PerStBiRo system returned to its zero point. If no reaction was detected, it automatically returned to the zero point after three seconds. The perturbation test ended with a 2-minute cool-down. The test was terminated before reaching the highest angle if participants held onto the handlebars repeatedly or reported feeling very unsteady and wanted to stop on their own.

**Fig. 2:**
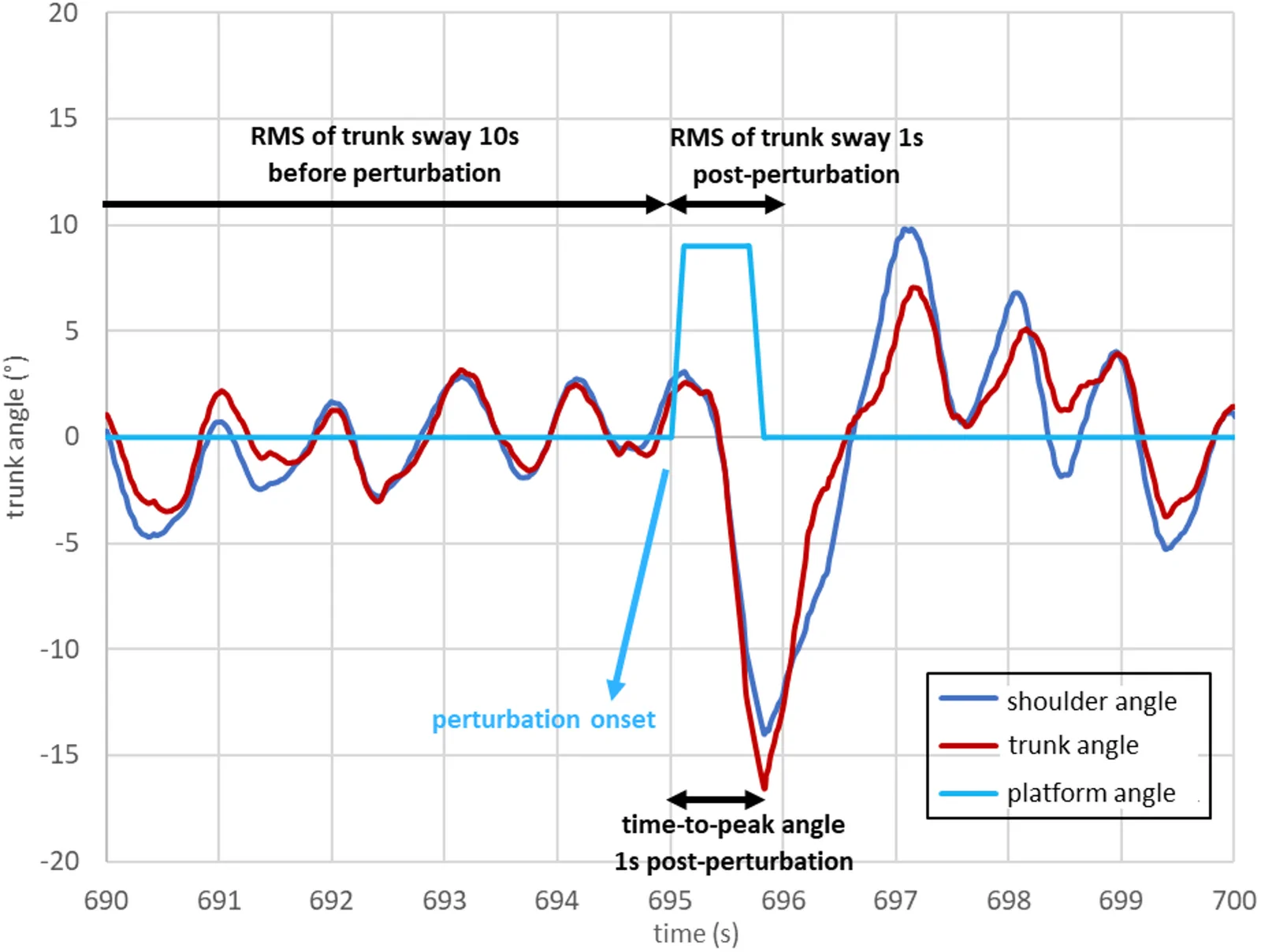
9° right tilt (positive angle) perturbation. A graph created from the saved CSV file. The system tilt angle increases (perturbation onset), followed by an onset of balance recovery reaction as seen by the trunk and shoulder angle. ML trunk time-to-peak angle is the time from perturbation onset to trunk peak angle within 1-second post-perturbation. Immediately after an effective balance recovery response was detected by the software, the PerStBiRo system returned to its initial horizontal condition. **Note**: UPC period is represented by small ML trunk and shoulder tilts from normal pedaling movements prior to the perturbation onset (10-second interval)

Figure 2 shows the dynamic cycling-specific stability during unperturbed cycling (UPC) that was quantified as the average ML root mean square (RMS) of trunk sway (°) over 10-second intervals before each perturbation. Reactive balance parameters to perturbed cycling (PC) included: (1) ML trunk time-to-peak angle – defined as the time from perturbation onset to trunk peak angle within 1-second post-perturbation; and (2) RMS of trunk sway (°) – defined as the RMS of angles within 1-second post-perturbation. We focused exclusively on perturbations with a platform tilt angle of at least 5°, as perturbations below this threshold did not consistently evoke detectable balance reactions, limiting the reliability of their identification.

### Descriptive measures and covariates

Demographic data such as sex, age, country, and living situation were collected. Body height was assessed using a wall-mounted stadiometer (seca 213; seca GmbH & Co. KG, Hamburg, Germany), and body weight was measured on the seca mBCA 514 (seca GmbH & Co. KG, Hamburg, Germany). Body Mass Index (BMI) was then computed. Concerns about falling were investigated using the Short Falls Efficacy Scale International (FES-I) [47]. Distance cycled per week was self-reported using predefined response options < 5 km, 5–10 km, 10–20 km, > 20 km.

### Data and statistical analysis

Means and standard deviations were calculated for metric parameters, and categorical variables were presented as percentages. Country differences were assessed using the Mann-Whitney U test and unpaired t-test for continuous and chi-squared test for categorical variables. Five persons had missing data: two did not perform the chair rise test, two stopped the perturbation test before reaching a perturbation angle of 5°, and one had missing data for both tests. These individuals were excluded from the analyses that required this information. Therefore, total sample size was reduced by less than 5% (5 out of 116). The main outcome is errors made in a standardized outdoor cycling course. As normality assumptions were not met for the cycling errors (Shapiro-Wilk test), nonparametric Spearman rank correlations between cycling errors and the balance tasks were examined to test bivariate relationships The subsequent explorative multiple regression analysis aimed to examine independent associations between cycling errors and balance performance while controlling for one another. First, a full multivariable regression model including all balance-related variables and the control variables age, sex, country and weekly distance cycled was computed. Variables showing statistical significance were subsequently entered into a final multivariable regression model, again adjusted for age, sex, country and weekly distance cycled. As sensitivity analysis, the final multivariable regression model was run again separately for both countries. Rather than relying on a predefined, hypothesis-driven selection of independent relations with the main outcome, potentially relevant balance-related variables were explored simultaneously. Prior to the analyses, data were checked for normality (Q-Q-plots, histograms) and outliers (standardized residuals, Cook’s distance). Multicollinearity was checked by the variance inflation factor. Correlation magnitude was interpreted as weak ≥ 0.10; moderate ≥ 0.30 and strong ≥ 0.50 [48]. A significance level of *α* = 0.05 was used for the entire analysis. The calculations were carried out using IBM SPSS Statistics version 31.0.0.0 (IBM Corp., Armonk, NY, USA).

## Results

### Sociodemographic and balance-related characteristics

Data of 116 participants (Israel: *n* = 43 (37.1%), Germany: *n* = 73 (62.9%)) aged 74.0 ± 5.4 years on average were included in the analysis, of whom 62 (53.4%) were women. Average errors in the cycling course were 13.1 ± 8.0. Average total SPPB score was 11.2 ± 0.9 and 14.5 ± 4.8 errors were recorded in the MBESS. Average gait speed was 1.1 ± 0.2 m/s and average time in the FSST was 9.6 ± 1.7 s. Errors in the cycling course were weakly associated with weekly distance cycled (r_s_ = -0.240, *p* = 0.009). A further description of the sample, differentiated by country, is shown in Table 1.

**Table 1:** Sociodemographic and balance-related characteristics of all (*N* = 116) older adults in the sample, differentiated by country

Characteristics	Total (*N* = 116): Mean *±* SD (range) *N* (%)	Israel (*n* = 43; 37.1%): Mean *±* SD (range) *N* (%)	Germany (*n* = 73; 62.9%): Mean *±* SD (range) *N* (%)
Age [years]^$^	74.0 ± 5.4 (65–87)	73.1 ± 4.8 (66–84)	74.6 ± 5.7 (65–87)
Women^+^	62 (53.4)	16 (37.2)**	46 (63.0)**
Living at home^+^	114 (98.3)	43 (100.0)	71 (97.3)
Body height [cm]^$^	167.6 ± 8.7 (149.5–195.0)	167.7 ± 8.2 (149.5–183.0)	167.6 ± 9.1 (150.0–195.0)
Body weight [kg]^$^	74.6 ± 15.8 (45.0–120.0)	75.7 ± 14.3 (45.0–109.0)	73.9 ± 16.6 (46.0–120.0)
Body mass index [kg/m²]^$^	26.4 ± 4.5 (17.6–44.1)	26.8 ± 3.9 (18.0–34.3)	26.2 ± 4.8 (17.6–44.1)
FES-I short form [7–28]^#^	8.4 ± 1.7 (7–18)	8.2 ± 1.6 (7–13)	8.6 ± 1.8 (7–18)
Distance cycled/week [category]^+^	2.0 ± 1.1 (1–4)	2.0 ± 1.2 (1–4)	2.0 ± 1.1 (1–4)
<5 km [category 1]	52 (44.8)	20 (46.5)	32 (43.8)
5–10 km [category 2]	27 (23.3)	11 (25.6)	16 (21.9)
10–20 km [category 3]	19 (16.4)	4 (9.3)	15 (20.5)
>20 km [category 4]	18 (15.5)	8 (18.6)	10 (13.7)
Cycling errors [0–57]^#^	13.1 ± 8.0 (3–34)	15.4 ± 8.7 (3–34)*	11.7 ± 7.3 (3–33)*
SPPB balance score [0–4]^#^	3.9 ± 0.4 (2.0–4.0)	3.9 ± 0.5 (2.0–4.0)	3.8 ± 0.4 (2.0–4.0)
SPPB total score [0–12]^#^	11.2 ± 0.9 (8–12)	11.4 ± 1.0 (8–12)	11.1 ± 0.9 (9–12)
MBESS total errors [0–30]^#^	14.5 ± 4.8 (3–26)	15.2 ± 4.8 (3–26)	14.0 ± 4.9 (4–20)
Gait speed [m/s]^$^	1.1 ± 0.2 (0.7–1.5)	1.0 ± 0.1 (0.7–1.3)***	1.1 ± 0.2 (0.8–1.5)***
FSST fastest time [s]^$^	9.6 ± 1.7 (6.0–16.4)	8.8 ± 1.6 (6.4–14.0)***	10.0 ± 1.6 (6.0–16.4)***

**abbreviations**: *SD* standard deviation; *FES-I* Falls Efficacy Scale – International Version; *SPPB* Short Physical Performance Battery; *MBESS* Modified Balance Error Scoring System; *FSST* Four Square Step Test. Country differences were assessed using the Mann-Whitney U test (^#^) and unpaired t-test (^$^) for continuous and chi-squared test (^+^) for categorical variables. Statistically significant difference between countries: *p* < 0.05*, *p* < 0.01**, *p* < 0.001***

### Bivariate correlation analysis

#### Static balance

A Spearman correlation between cycling errors and SPPB side-by-side and semi-tandem stance could not be computed because the ordinal variables were constant across participants. Further, three participants were not able to do the repeated chair rise and were excluded from this analysis. No significant associations were found between cycling errors and the SPPB tandem feet (r_s_ = -0.145, *p* = 0.121), the SPPB balance score (r_s_ = -0.145, *p* = 0.121) and the SPPB total score (r_s_ = -0.153, *p* = 0.106). Time in SPPB chair rise was not significantly correlated either (r_s_ = 0.130, *p* = 0.170). More errors in the cycling course were weakly associated with more errors for total MBESS (r_s_ = 0.213, *p* = 0.022), MBESS single-leg stance (r_s_ = 0.205, *p* = 0.027) and MBESS tandem feet (r_s_ = 0.186, *p* = 0.046), with no significant correlation for MBESS side-by-side (r_s_ = -0.011, *p* = 0.907) (Table 2).

**Table 2:** Spearman correlations between outdoor cycling errors and static balance function

	Outdoor cycling errors score		
	*r*_s_	*p*	*N*
**SPPB**			
SPPB total score	-0.153	0.106	113
SPPB balance score	-0.145	0.121	116
SPPB side-by-side	–	–	–
SPPB semi-tandem	–	–	–
SPPB tandem feet	-0.145	0.121	116
SPPB chair rise [s]	0.130	0.170	113
**MBESS**			
MBESS total errors	0.213*	0.022	116
MBESS side-by-side	-0.011	0.907	116
MBESS single-leg	0.205*	0.027	116
MBESS tandem feet	0.186*	0.046	116

r_*s*_ Spearman’s rank correlation coefficient; **abbreviations**: *SPPB* Short Physical Performance Battery; *MBESS* Modified Balance Error Scoring System. Statistically significant association: *p* < 0.05*

#### Dynamic balance and unperturbed indoor cycling

More cycling errors were weakly associated with slower gait speed (r_s_ = -0.203, p = 0.029), but not with the time in the FSST (rs = 0.028, p = 0.764). Ten participants (8.6%) asked to stop the perturbation test before reaching the highest level. Three participants (2.6%) were excluded from the analysis of the PerStBiRo data, because they stopped before reaching a perturbation angle of 5°. More errors in the cycling course were weakly associated with higher average RMS of trunk sway during 10-seconds intervals of UPC prior to each perturbation (r_s_ = 0.246, *p* = 0.009) (Table 3).

**Table 3:** Spearman correlations between outdoor cycling errors and dynamic balance

	Outdoor cycling errors score		
	*r*_s_	*p*	*N*
Gait speed [m/s]	-0.203*	0.029	116
FSST [s]	0.028	0.764	116
**Perturbation test – UPC**			
Unperturbed RMS of trunk sway [°]	0.246**	0.009	113

r_s_ Spearman’s rank correlation coefficient; **abbreviations**: *FSST* Four Square Step Test; *UPC* Unperturbed cycling; *RMS* Root Mean Square. Statistically significant association: *p* < 0.05*, *p* < 0.01**

#### Perturbed indoor cycling reactive balance

More errors in the cycling course were weakly associated with less RMS of trunk sway within 1-second post-perturbation (r_s_ = -0.295, *p* = 0.002) and faster trunk reaction time-to-peak angle (r_s_ = -0.289, *p* = 0.002) (Table 4).

**Table 4:** Spearman correlations between outdoor cycling errors and reactive balance

	Outdoor cycling errors score		
	*r*_s_	*p*	*N*
**Perturbation test – PC**			
Perturbed RMS of trunk sway [°]	-0.295**	0.002	113
Trunk time-to-peak angle [s]	-0.289**	0.002	113

r_s_ Spearman’s rank correlation coefficient. **abbreviations**: *PC* Perturbed cycling; *RMS* Root Mean Square. Statistically significant association: *p* < 0.05*, *p* < 0.01**

### Multiple regression analysis

A multiple linear regression analysis adjusted by age, sex country and weekly distance cycled showed that longer time in the FSST (B = 1.281, 95% CI [0.391, 2.170], *p* = 0.005) and higher average ML RMS of trunk sway during UPC over 10-second intervals before each perturbation (B = 2.685, 95% CI [0.602, 4.767], *p* = 0.012) were significant independent correlates of more cycling errors. The initial model explained 25.4% (adjusted R² = 0.254) and the final model 25.7% of the variance (adjusted R² = 0.257) (Table 5). As sensitivity analysis, the final model was run separately for both countries. Results were comparable for the German center (FSST: B = 1.411, 95% CI [0.380, 2.443], *p* = 0.008; unperturbed RMS of trunk sway: B = 2.518, 95% CI [0.247, 4.789], *p* = 0.030; adjusted R² = 0.188). However, the Israeli center yielded no statistically significant associations, despite comparable unstandardized regression coefficients and adjusted R² values relative to the main analysis (FSST: B = 1.231, 95% CI [-0.563, 3.028], *p* = 0.173; unperturbed RMS of trunk sway: B = 3.481, 95% CI [-1.327, 8.290], *p* = 0.151; adjusted R² = 0.250).

**Table 5:** Multiple linear regression analysis of balance performance and outdoor cycling errors (*N* = 111 for full model and *N* = 113 for final model, adjusted for sex, age, country and weekly distance cycled)

	Full model			Final model		
	B	*p*	B	SE	95% CI	*p*
SPPB total score	1.810	0.231				
SPPB balance score	-1.453	0.515				
SPPB chair rise [s]	0.630	0.291				
MBESS total errors	0.101	0.520				
Gait speed [m/s]	-5.616	0.267				
FSST [s]	1.148*	0.032	1.281**	0.448	[0.391, 2.170]	0.005
Unperturbed RMS of trunk sway [°]	2.614*	0.032	2.685*	1.050	[0.602, 4.767]	0.012
Perturbed RMS of trunk sway [°]	0.064	0.935				
Trunk time-to-peak angle [s]	-32.131	0.058				
**Model fit**						
Full model R² = 0.342; adjusted R² = 0.254; F(13, 97) = 3.877, *p* < 0.001						

Final model R² = 0.297; adjusted R² = 0.257; F(6, 106) = 7.469, *p* < 0.001

B unstandardized regression coefficient from multiple linear regression; **abbreviations**: *SE* standard error; *CI* confidence interval; *SPPB* Short Physical Performance Battery; *MBESS* Modified Balance Error Scoring System; *FSST* Four Square Step Test; *RMS* Root Mean Square. Statistically significant association: *p* < 0.05*, *p* < 0.01**

## Discussion

The aim of this exploratory study was to investigate the association between outdoor cycling competence, operationalized as the number of errors on a standardized cycling course, and static, dynamic, and reactive trunk balance control during the PerStBiRo perturbation test in community-dwelling older adults. No bivariate associations were found between outdoor cycling errors and SPPB score and time in the FSST. Yet, more outdoor cycling errors were weakly associated with more errors in MBESS, slower gait speed and the laboratory cycling balance test (higher RMS of trunk sway during UPC, faster trunk time-to-peak angle, and lower RMS of trunk sway during PC). After adjusting for age, sex and country, the final model (adjusted R² = 0.257) of the multivariable regression analysis revealed that only time in the FSST and RMS of trunk sway during UPC remained independently associated with cycling errors, both measures of dynamic balance control.

To better contextualize the number of errors made in the cycling course, a comparison with data from the German SiFAr trial [18, 49] was conducted. In BiPerAge, participants made on average 13.1 ± 8.0 errors in the cycling course. In the SiFAr trial, on average 9.1 ± 7.4 errors were made at baseline [18]. It should be noted that in BiPerAge, the cycling course was made more challenging by modifying two stations, as a ceiling effect was found in the SiFAr trial [18]. Additionally, only participants with at least three errors in the cycling course were enrolled in the current study while the SiFAr trial also included persons with less than three errors [18]. This selection may also explain in part the higher error scores in the BiPerAge study.

In the present study, participants exhibited relatively high functional performance, reflected by their high SPPB scores (11.2 ± 0.9, range 8–12) indicating low risk of mobility disability [38]; relatively fast FSST (9.6 ± 1.7s, range 6.0–16.4), typically below 10 s, indicating healthy, non-frail older adults without fall history [50, 51]; an average gait speed above 1 m/s (1.1 ± 0.2 m/s, range 0.7–1.5) indicates good functionality and has been associated with survival [52–54], and little to no concerns about falling [52, 53, 55]. The high functional ability and minor variance between the participants may have contributed to the absence of association in the bivariate correlation analysis between SPPB scores and cycling competence, likely due to a ceiling effect in SPPB that reduced its sensitivity to detect differences in cycling-related capabilities.

Static balance performance was then additionally examined using the more challenging MBESS [41, 56]. Both MBESS and SPPB include standing balance tasks (side-by-side, semi-tandem, tandem), however, MBESS places higher demands on participants’ postural stability than SPPB due to the eyes-closed condition. It may have greater potential to detect balance dysfunction in older adults with good functional performance. Yet, no association was found between cycling errors and the side-by-side stance in MBESS. The tandem stance and single-leg stance, which impose greater ML balance demands, showed weak associations. We assume that cycling and basic standing balance tasks may not represent identical motor tasks. However, the associations found in the challenging MBESS tandem stance and single-leg stance may suggest that these tasks partially share underlying postural control mechanisms with outdoor cycling. Previous investigations found that older cyclists maintained the single-leg stance longer than non-cyclists [57, 58]. Single-leg stance is a well-established measure for ML balance in older adults [26]. The bivariate associations may suggest that more challenging standing balance tasks could better reflect cycling-relevant postural control mechanisms in older adults (i.e., cycling ML trunk sway control). However, the effect sizes were only weak for all tasks.

Dynamic stability was assessed using the FSST and usual gait speed examination. In the bivariate correlation analysis, we found a weak negative association of gait speed with errors in the cycling course, but no correlation for time in the FSST. Especially gait speed is an important indicator of overall health in older adults [52–54] and the association may indicate that a good functional status co-occurred with better performance in the cycling course. Since the effect of age on functional performance cannot be ignored [59], age was included in the subsequent regression analysis.

In addition, we found a weak association between the RMS of trunk sway during UPC and cycling competence which represents dynamic cycling-specific ML trunk control. This indicates that measures incorporating dynamic and task-specific balance demands may be useful to capture the motor abilities required for successful cycling competence. It must be taken into account that in less demanding balance tasks, the lower body, especially ankle and hip muscles, primarily ensures the whole body stability [8]. However, in more challenging tasks, the upper body, especially the trunk, seems to play an additional important role in balance performance and stability [21, 23, 60, 61]. In a task such as cycling, that comes with a high balance requirement and where the seated position may reduce the contribution of leg and hip musculature, we assume that the trunk plays an important role in compensatory ML movements when riding through curves, having to brake abruptly or encountering uneven ground [25]. Research in this area is still scarce, but a study of younger, fit cyclists showed that more skilled cyclists relied on their lean control with smaller inputs to maintain control while cycling on a roller [24]. Along with this result, an analysis of older cyclists and non-cyclists suggested that under safe and less challenging conditions, cyclists used a more tightly regulated balance control strategy, reflected in less ML postural sway [57]. This finding supports our assumption that ML trunk control and cycling errors may be linked, which was further examined in the regression model discussed below.

Further, bivariate analyses showed weak negative associations between errors in the outdoor cycling course and reactive time-to-peak ML trunk angle as well as ML RMS of trunk sway during PC. Greater ML trunk sway during PC in the bivariate analysis, accompanied by a longer time-to-peak trunk angle within 1-second post-perturbation may reflect a more flexible or responsive postural control strategy that allows greater movement excursion during cycling and recovery from unexpected perturbations, possibly indicating a greater capacity to react to balance perturbation. As stated above, it was found that older cyclists showed less postural sway under subjectively safer balance conditions than non-cyclists [24, 57]. Under more challenging conditions, like performing the perturbation test, it may be that older adults with better cycling competence implement a different strategy. A previous study suggested that a greater range of ML motion during challenging tasks was associated with greater ML stability and control in cyclists [57]. Skilled older cyclists may have reacted to perturbations with greater confidence and smoother movement patterns due to better ML balance and use of trunk control strategy [25].

Using multiple regression models, we exploratorily estimated the independent relationship between errors in the cycling course and each balance instrument while controlling for age, sex, country and weekly distance cycled. We did not find significant independent associations of SPPB score or MBESS errors in the fully adjusted regression model. We hypothesize that, as both are instruments measuring mainly static balance, they may not sufficiently explain the more dynamic demands of cycling. Regarding the dynamic measurements, time in the FSST and RMS of ML trunk sway during UPC emerged as significant independent correlates of cycling errors, while gait speed was not independently associated, which may be explained by its dependence on age [54]. In a sensitivity analysis, this result could be confirmed for the German center. However, the Israeli center yielded no statistically significant associations, despite comparable unstandardized regression coefficients and adjusted R² values relative to the main analysis. This discrepancy may be attributable to insufficient statistical power, as only 43 Israeli participants were analyzed compared with 70 participants from Germany. The FSST is a complex instrument requiring whole-body dynamic stability and interlimb coordination [45, 46]. A successful performance requires dynamic, rapid, multidirectional weight shifts, changes in direction, trunk stability and stabilization of the body’s center of mass [45, 46]. Its independent association only became apparent after adjustment for the included confounders, as the relationship was obscured in the bivariate analysis by differences between men and women. Although women completed the FSST faster, they also made more errors in the cycling course, resulting in a suppression of the underlying association. For this reason, the association between the FSST and cycling competence should be examined by sex. Performance in the FSST seemed to demonstrate effective balance control including precise directional anticipated weight bearing changes and stepping, that appear to be linked independently to cycling errors in older adults. Trunk sway during UPC seemed to be transferable to good outdoor cycling competence as well, even though the perturbation test was performed in an upright position without holding onto the handlebars. The reactive parameters measured during PC were not independent correlates. The dynamic sway during UPC on the PerStBiRo system may have more realistically simulated steady, stable driving on the cycling course, while perturbations may have primarily demonstrated short-term reactive capacity, which may not translate directly to the continuous unperturbed tasks of the outdoor cycling course. Accordingly, UPC appears to provide a realistic proxy of the continuous dynamic balance control necessary for outdoor cycling. However, PC provoked isolated reactive postural responses for 1-second intervals, that may not capture the predictable balance challenges of the cycling course (e.g., no sudden unexpected reactions were required). Reaction patterns in seconds two and three following a perturbation should be taken into account in future analyses to better capture the sway pattern following perturbation and other compensatory movements to see if the re-stabilization phase might better reflect the cycling course demands.

Taken together, the present findings suggest that the relationship between balance and cycling competence might be domain-specific. In the final regression model, dynamic balance and cycling-specific trunk control during UPC exhibited weak associations with outdoor cycling competence. Assessments of older cyclists may benefit from incorporating challenging dynamic balance measures, such as the FSST, together with task-specific evaluations of trunk control during cycling. Such approaches may provide greater insight into the balance mechanisms underlying successful cycling performance and may help identify individuals at risk for cycling-related difficulties. However, future studies should further examine these relationships using longitudinal analyses to draw more reliable statements.

Certain limitations of the study should be mentioned. Apart from the PerStBiRo system, there is currently no comparative test for measuring bicycle-specific dynamic and reactive balance indoors. Nevertheless, the system has proven to be feasible so far [34, 35]. Backward and forward perturbations were excluded from the analysis since the reaction angles showed no linear association to the perturbation level. Additionally, as we intended to create a holistic view of the balance abilities of older cyclists, it has to be mentioned that we did not include instruments for assessing upper body static balance, and conversely, lower body reactive balance. In addition, balance assessment was not quantified using inertial measurement units or motion capture (only in the PerStBiRo system). Regarding physical activity levels, only weekly distance cycled was surveyed. Further, participant inclusion was conducted throughout the year, and weather effects in assessing weekly distance cycled and the errors in the cycling course may have occurred [62]. Overall, the sample consisted of older adults of good functional status while experienced cyclists, who had less than three errors in the cycling course, were excluded from the study. Participants with vestibular conditions were included if they were able to perform the cycling course at baseline. This medical issue might have affected participants’ balance. Caution should be exercised when generalizing the results to other populations. Finally, the cross-sectional design does not permit conclusions regarding causal mechanisms. As this study was exploratory in nature, no formal a priori sample-size or precision justification was performed. We acknowledge an increased risk of Type I error inflation due to multiplicity. Consequently, all regression results must be interpreted with caution as exploratory and hypothesis-generating rather than confirmatory. Further, it might be argued that older adults who perform well on the cycling course are generally fitter and would also perform well on other measures of balance. However, only balance measures that mostly required trunk-related dynamic control were significantly associated with cycling course performance, whereas balance measures that imposed relatively low demands on trunk-related balance control showed no significant associations. This pattern may suggest that the observed relationships are task-specific and are not merely a reflection of superior overall balance ability.

The study has multiple strengths. The associations of outdoor cycling errors in older adults and all three components of balance control (static, dynamic and reactive) considered in this cross-sectional analysis have never been investigated before. Furthermore, the PerStBiRo system is a unique and feasible tool for assessing cycling-specific dynamic and reactive balance [34, 35]. The BiPerAge trial is the first project using the PerStBiRo system as a training tool to investigate its influence on outdoor cycling competence and to examine older cyclists from Israel and Germany. Objectively assessing outdoor cycling competence using the cycling course [18, 36] is valuable for determining the relationship between the balance tests conducted in the laboratory and real-world performance. The variety of selected balance instruments provided a comprehensive picture of the participants’ overall balance control and functional status.

## Conclusions

We assessed associations between outdoor cycling errors and static, dynamic as well as reactive balance function in older adults. Exploratory multiple regression analysis revealed that dynamic balance tasks involving a trunk-related balance component, represented as time in the FSST and ML trunk sway during UPC on the PerStBiRo system, were weakly independently associated with cycling competence, whereas static and reactive measures were not. It may therefore be possible to identify a movement profile among older cyclists that is associated with better cycling competence. Older adults who show effective dynamic trunk-related balance control in challenging dynamic tasks may score fewer errors in the cycling course. Additionally, a more tightly regulated trunk sway during UPC was associated with better results in the cycling course. Therefore, assessments of older cyclists may benefit from incorporating challenging dynamic balance measures, such as the FSST, together with task-specific evaluations of trunk control during cycling. Future research could benefit from this understanding of the movement patterns of older cyclists, but must explore these weak relationships in greater depth using longitudinal analyses to draw more reliable statements.

## Supplementary Information

Below is the link to the electronic supplementary material.

Supplementary Material 1

Supplementary Material 2

## Data Availability

The datasets generated during this study are not publicly available, but are available from the corresponding author on reasonable request.

## Abbreviations

BiPerAge: The Transfer Effects of Stationary Bicycle Perturbation Training on Older Adults’ Cycling Skills and Behavior
BMI: Body Mass Index
FES-I: Short Falls Efficacy Scale – International Version
FSST: Four Square Step Test
ICC: Intraclass Correlation Coefficient (ICC)
MBESS: Modified Balance Error Scoring System
ML: Mediolateral
PBBT: Perturbation-based balance training
PC: Perturbed cycling
PerStBiRo: Perturbation Stationary Bicycle Robotics
RMS: Root mean square
SiFAr: Safer Cycling in Older Age
SPPB: Short Physical Performance Battery
UPC: Unperturbed cycling
