# Supplementary material for: Outdoor cycling competence and balance control in older adults – cross-sectional analysis of the BiPerAge trial
Source: BMC Geriatr. 2026 Oct 19;26:1306. doi: 10.1186/s12877-026-08491-7 (PMC13682123; doi:10.1186/s12877-026-08491-7)
Supplement: Supplementary file 1 [file 12877_2026_8491_MOESM1_ESM.docx]

**Manual cycling course**

Inhalt

[1.	Requirement	2](#_Toc229135573)

[2.	Layout cycling course	3](#_Toc229135574)

[3.	General information	4](#_Toc229135575)

[4.	Execution	5](#_Toc229135576)

[5.	Instructions	11](#_Toc229135577)

[6.	Analysis	15](#_Toc229135578)

1. Requirements

Equipment for the cycling course:

- 2x measuring tape
- Chalk spray
- Rope (orientation for spraying)
- "Circle" for spraying figure eight
- 6x traffic cones
- Flat cone (last cone of the slalom station)
- 2x hoops
- 2x stopwatches
- 2x cameras
- 2x tripods (120-140 cm height)
- 2x protocol sheets per participant
- 2x pencils
- 2x clipboards
- Numbers for station turning to the left (0-7)

At least 3x test supervisors are necessary:

- Equipment for test supervisor 1:
  - Protocol sheet
  - Clipboard
  - Pencil
  - Stopwatch
- Equipment for test supervisor 2:
  - Protocol sheet
  - Clipboard
  - Pencil
  - Camera 2
- Equipment for test supervisor 3:
  - Numbers for station turning to the left (0-7)
  - Camer

2. Layout cycling course

3. General information

- Setup
  - Standardized positioning of cameras:
    - Set up camera 1 in line with the “Slow cycling” corridor.
    - Set up camera 2 in line with the “Narrow alley”.
- Instructions
  - Before video recordings are made of participants, ensure that consent for video recording has been provided.
  - Each participant walks through the cycling course and gets a briefing about the respective stations, then completes a practice run. Only then the actual testing takes place.
  - Observe the test person's exercise run closely and then point out errors (e.g. omission of a cone) as well as correct execution (e.g. participants must remain seated on the bike while riding through the cycling course) as detailed as possible. However, do not offer any strategies for " better" execution of the respective stations (e.g. picking up pace before/in the station "Narrow alley").
  - For detailed instructions see below.
- Test execution
  - Detection of the right error category (e.g. touching a cone).
  - Ensure that the documentation is correct and as complete as possible.
    - The measuring point on the bike is always the tire-ground contact point and not the start of the front wheel (i.e. not like a light barrier measurement). Especially important for time measurement at "Slow cycling" (station 2) or “Touching the line”.
  - Errors (see protocol cycling course below):
    - **Prioritization of the documentation of certain error categories during the course** (Some errors are not always clearly visible in the camera recording).
    - Prioritization of the documentation of the error frequency of certain error categories regarding multiple errors (e.g. 3 cones were touched).
    - Follow-up errors “Touching the line”:
      - Touching the lines/touching the cones/hoop with the bike: If the rear tire touches a line/touches a cone/hoop directly after the front tire, this is only considered as one error (follow-up error).
    - Errors are only registered within the individual stations:
      - No documentation between stations 1-2
      - No documentation between stations 3-4
      - No documentation between stations 5-6

4. Execution

**Before the start:**

Procedure/guidelines for test supervisor 1:

- Query the participant's readiness to start.
- Give recording signal to camera operators.
- Give the start signal to the participant.

Procedure/guidelines for test supervisor 2:

- Signalizing readiness for recording (camera 2).
- Camera 2 must be set to slalom at the start of recording.
- When participant passes station 3 d), camera 2 must be aligned with narrow alley.
- After participant passes turning left, camera must be aligned with figure 8.
- After repositioning of camera 2, test supervisor 2 supports with error protocolling during figure 8.

Procedure/guidelines for test supervisor 3:

- Signalizing readiness for recording (camera 1).
- Camera 1 must be set to slalom at the start of recording.
- When participant passes station 3 b), camera 1 must be aligned with turning to the left
- After repositioning of camera 1, test supervisor 3 goes to the X and presents the number for station “Turning to the left”.

**1. Station: Slalom:**

| Procedure/guidelines for participant | Error category |
| --- | --- |
| Avoiding contact with the cones | Touching of cones |
| Cycling around all cones | Omission of cones |
| Avoiding foot-to-floor contact | Foot-to-floor contact |
| Passing the slalom sitting on the saddle | Cycling in a standing position |
| Slalom exit |  |
| Possible rest before station 2 |  |

Procedure/guidelines for test supervisor 1:

- Try to stay at the same level as the participant.
- Provide enough space for the turns of the participant.
- Avoid the angle of view of the cameras.
- Prioritization during this station:
  - **Touching of cones** should be accurately documented (sometimes not clearly visible in the video).
  - **Foot-to-floor contact**: The number of contacts should be accurately documented (sometimes not clearly visible in the video).

**2. Station: Slow cycling (Timed by stopwatch):**

| Procedure/guidelines for participant | Error category |
| --- | --- |
| Avoiding crossing the sideline | Touching the sideline |
| Avoiding foot-to-floor contact | Foot-to-floor contact |
| Passing the corridor sitting on the saddle | Cycling in a standing position |
| Passing the corridor in at least 10 sec | Time error (x < 10 sec) |
| No rest before station 3 possible |  |

Procedure/guidelines for test supervisor 1:

- Time measurement starts/ends when crossing the start/end line of the corridor with the front tire (tire-ground contact).
- Walk at a constant pace, next to the corridor and diagonally in front of the participant, to the corridor finish line within 10 seconds (=pacemaker for the participant).
- After the station: Participants should continue directly to station 3.
- Prioritization during this station:

1. **Timekeeping** at contact point tire-ground (not clearly visible in the video).
2. **Pacemaker:** Ensure constant gait speed.

**3. Station a): Dismounting right:**

| Procedure/guidelines for participant | Error category |
| --- | --- |
| Approaching the hoop while seated | Cycling in a standing position |
| Stopping left of the hoop |  |
| Right foot is placed directly from the pedal into the hoop | Foot-to-floor contact outside of the hoop (both feet)/ Touching the hoop with the foot/ bicycle |
| Left foot is placed directly from the pedal into the hoop |  |

Procedure/guidelines for test supervisor 1:

- Try to stay at the same level as the participant.
- Provide enough space for the participant.
- Avoid the angle of view of the cameras.
- Prioritization during this station:
  - **Touching of the hoop** should be accurately documented (sometimes not clearly visible in the video).
  - **Foot-to-floor contact**: The number of contacts should be accurately documented (sometimes not clearly visible in the video).

**3. Station b): Mounting right:**

| Procedure/guidelines for participant | Error category |
| --- | --- |
| Left foot is placed directly from the hoop onto the outer pedal | Foot-to-floor contact outside of the hoop (both feet)/ Touching the hoop with the foot/ bicycle |
| Momentum is gained with the right foot inside the hoop |  |
| Right foot is placed directly from the hoop onto the inside pedal |  |
| Cycling to the next hoop sitting on the saddle |  |
| No rest before station 3c) possible |  |

Procedure/guidelines for test supervisor 1:

- Try to stay at the same level as the participant.
- Provide enough space for the participant.
- Avoid the angle of view of the cameras.
- Prioritization during this station:
  - **Touching of the hoop** should be accurately documented (sometimes not clearly visible in the video).
  - **Foot-to-floor contact**: The number of contacts should be accurately documented (sometimes not clearly visible in the video).

Procedure/guidelines for test supervisor 3:

- After station 3b, camera 1 must be aligned with “Turning to the left”
- After repositioning of the camera, go to the **X** and present the number.

**3. Station c): Dismounting left**

| Procedure/guidelines for participant | Error category |
| --- | --- |
| Approaching the hoop while seated | Cycling in a standing position |
| Stopping right of the hoop |  |
| Left foot is placed directly from the pedal into the hoop | Foot-to-floor contact outside of the hoop (both feet)/ Touching the hoop with the foot/ bicycle |
| Right foot is placed directly from the pedal into the hoop |  |

Procedure/guidelines for test supervisor 1:

- Try to stay at the same level as the participant.
- Provide enough space for the participant.
- Avoid the angle of view of the cameras.
- Prioritization during this station:
  - **Touching of the hoop** should be accurately documented (sometimes not clearly visible in the video).
  - **Foot-to-floor contact**: The number of contacts should be accurately documented (sometimes not clearly visible in the video).

**3. Station d): Mounting left:**

| Procedure/guidelines for participant | Error category |
| --- | --- |
| Right foot is placed directly from the hoop onto the outer pedal | Foot-to-floor contact outside of the hoop (both feet)/ Touching the hoop with the foot/ bicycle |
| Momentum is gained with the left foot inside the hoop |  |
| Left foot is placed directly from the hoop onto the inside pedal |  |
| Continue cycling sitting on the saddle |  |
| Possible rest before station 4 |  |

Procedure/guidelines for test supervisor 1:

- Try to stay at the same level as the participant.
- Provide enough space for the participant.
- Avoid the angle of view of the cameras.
- Prioritization during this station:
  - **Touching of the hoop** should be accurately documented (sometimes not clearly visible in the video).
  - **Foot-to-floor contact**: The number of contacts should be accurately documented (sometimes not clearly visible in the video).

Procedure/guidelines for test supervisor 2:

- After station 3 d, camera 2 must be aligned with narrow alley.
- After station left turning, camera 2 must be aligned with figure 8.
- Then support with assessing errors in figure 8.

**4. Station: Narrow alley:**

| Procedure/guidelines for participant | Error category |
| --- | --- |
| Avoiding foot-to-floor contact | Foot-to-floor contact |
| Avoiding crossing the sideline | Touching the sideline |
| Passing the alley sitting on the saddle | Cycling in a standing position |
| No rest before station 5) Possible |  |

Procedure/guidelines for test supervisor 1:

- Try to stay at the same level as the participant.
- Provide enough space for the participant.
- Avoid the angle of view of the cameras.
- Prioritization during this station:
  - **Touching of the sideline** should be accurately documented (sometimes not clearly visible in the video).

**5. Station:** **Turning to the left:**

| Procedure/guidelines for participant | Error category |
| --- | --- |
| Correct hand signal | No hand signal/ Hand signal too late |
| Correct shoulder check | No shoulder check |
| Recognizing the number | No/incorrect naming of the number |
| Avoiding crossing the sideline | Touching the sideline |
| Avoiding foot-to-floor contact | Foot-to-floor contact |
| Passing the corridor sitting on the saddle | Cycling in a standing position |
| Turning to the left within the corridor |  |
| Possible rest before station 6 |  |

Procedure/guidelines for test supervisor 1:

- Try to stay at the same level as the participant.
- Provide enough space for the turn of the participant.
- Avoid the angle of view of the cameras.
- Prioritization during this station:
  - **Touching of the sideline** should be accurately documented (sometimes not clearly visible in the video).
  - **Shoulder check** should be accurately documented (sometimes not clearly visible in the video).

Procedure/guidelines for test supervisor 2:

- After participant passes turning left, camera 2 must be aligned with figure 8.
- After repositioning of camera 2, test supervisor 2 supports with error documentation in figure 8.

Procedure/guidelines for test supervisor 3:

- Presentation of the number at chest height until the participant reaches the turn.
- When the participant starts the turning process, the number is no longer presented.
- Then support with error documentation in figure eight.

**6. Station: Cycling a figure eight:**

| Procedure/guidelines for participant | Error category |
| --- | --- |
| Following the arrow into the circle structure |  |
| Avoiding foot-to-floor contact | Foot-to-floor contact |
| Avoiding crossing the sideline | Touching the sideline |
| Following the arrows, cycle one figure eight | No eight on the first attempt |
| Following the arrows, cycle in a seated position | Cycling in a standing position |

Procedure/guidelines for test supervisor 1:

- Try to stay at the same level as the participant.
- Provide enough space for the turns of the participant.
- Avoid the angle of view of the cameras.
- Prioritization during this station:
  - **Touching of the sideline** should be accurately documented (sometimes not clearly visible in the video).

Procedure/guidelines for test supervisor 2:

- Try to stay at the same level as the participant.
- Provide enough space for the turns of the participant.
- Avoid the angle of view of camera 2!!
- Prioritization during this station:
  - **Touching of the sideline** should be accurately documented (sometimes not clearly visible in the video).

**After crossing the finish line:**

Procedure/guidelines for test supervisor 1:

- Inquiry of the number and documentation (cannot be heard on video recording).

5. Instructions

**General instructions:**

“First and foremost, your well-being is important to us and we want you to get through the cycling course unharmed. Therefore, you should try to follow the subsequent instructions only to the best of your abilities. Nevertheless, try to complete the individual stations as flawlessly as possible. Once you have started a station, try to finish it. You should not stop halfway through and start again. The total time required to complete the cycling course is irrelevant. You can theoretically take a break between certain stations (1-2; 3-4). Nevertheless, you should try to cycle through the course in one attempt if possible. We will now walk through the course together. I will explain the individual stations of the course to you. The course consists of six different stations.”

**Station 1 (Slalom):**

“The first station is about cycling around the cones in a slalom at a self-selected pace. Please try to enter the slalom following the arrow. The gaps between the cones get increasingly smaller. Please do not ride around the cones standing up and remain seated in your saddle. Try not to touch the cones when passing them. We have replaced the last cone with a flat cone that can simply be run over if necessary.” (Walk the course with the participant).

“After that, you take a left turn, the lines are only for orientation. You don't have to ride exactly along the line. You can take a break here if necessary and make a loop.” (Walk the course with the participant).

**Station 2 (Slow cycling):**

“For the second station, I ask you to cycle as slowly as possible through the marked corridor. Try to pass through the corridor in at least 10 seconds. I will walk diagonally in front of you next to the corridor. When cycling through the corridor, you can orient yourself to the pace I set and try to stay behind me as well as possible. As soon as you cross the corridor line with the front tire, I will start the time measurement. As soon as you cross the second corridor line with the front tire, I will end the time measurement. Please remain seated in the saddle while cycling and try not to touch the side lines with your tires. You should then proceed directly to the third station.”

**Station 3 (Dismounting to both sides):**

“At the third station, I would like you to step off once to the right side and once to the left side into the hoops lying on the ground. For this purpose, please cycle up to the hoop in a seated position at a self-selected pace. The first time you stop, the hoop should be on the right side of the bike. Stop next to the hoop and step directly into the hoop with your right foot. Meanwhile, your left foot should rest on the pedal. Then lift your left leg over the bike and step into the hoop. Afterwards, lift your left leg over the bike again and place the foot directly on the left pedal. Try to create enough momentum with your right foot to step directly out of the hoop onto the right pedal to continue cycling in a seated position at a self-selected pace without touching the ground again. Please cycle until you reach the next hoop. The hoop should be on the left side of the bike. Stop next to the hoop and step directly into the hoop with your left foot. Meanwhile, your right foot should rest on the pedal. Then lift your right leg over the bike and step into the hoop. Afterwards, lift your right leg over the bike again and place your foot directly on the right pedal. Try to create enough momentum with your left foot to step directly out of the hoop onto the left pedal to continue cycling in a seated position at a self-selected pace without touching the ground again. Please try not to touch the tires with your feet or your bike during the entire process.” (Walk the course with the participant and pretend to step into the hoop).

“Afterwards you will take a right turn, the lines are again only for orientation. You do not have to ride exactly along the line. You can take a break here if necessary.” (Walk the course with the participant).

**Station 4 (Narrow alley):**

"At the fourth station, you are now asked to cycle through the marked corridor. Please try to remain seated in the saddle while cycling at a self-selected pace and avoid touching the side lines with your tires. You should then proceed directly to the fifth station and make sure that you do not cross the side lines there either.” (Walk the course with the participant).

**Station 5 (Turning to the left side):**

“At the fifth station, you are required to turn left (in compliance with the road traffic regulations). Cycle within the marked corridor at a self-selected pace, give a hand signal in due time and look over your left shoulder when you have passed the person standing next to the corridor. Please give the hand signal before you have started to make the turn. While looking over your shoulder, the person standing next to the corridor will hold up a piece of paper with a number on it. Please try to read the number displayed in due time and memorize it. When you start to make the turn, the number will be hidden again. Then turn left within the marked lines. Please try not to touch the side lines with either the front or rear tire and remain seated in the saddle while cycling at this station either. After completing the course, I will ask you for the number displayed. You should then proceed directly to the sixth and last station and make sure that you do not cross the side lines there either.” (Walk the course with the participant).

**Station 6 (Cycling a figure eight):**

“The last station is about cycling a figure eight. Please turn left into the circles following the arrow. Following the drawn arrows, cycle around the two circles once in the shape of an eight. Please try to complete the eight in a single attempt. Ride the 8 in one go and not first one circle and then the other. Please try to remain seated on the saddle while cycling at a self-selected pace and avoid touching the (outer and inner) side lines with your tires. Finally, take a left turn to the finish line. There I will ask you for the number displayed in station 5.”

**Concluding instructions:**

"Please cycle through the course in such a way that you feel safe on the bike at all times. If you feel unwell or dizzy during the course, please stop. It's not about speed, but about performing the individual stations as correctly as possible. The total time does not matter and, as I said, is not assessed. Have you understood the instructions for all the stations? If so, you can now cycle through the course once to practice. Afterwards I will give you feedback on whether you have made any mistakes at the respective stations. (Please put on your helmet.) If you have any further questions, please feel free to ask me.”

**Feedback after the test drive**

Then have participants carry out the test drive and give as detailed as possible feedback on the errors made. Point out errors, but do not offer any strategies for "better" execution of the respective stations (e.g. picking up pace in the station "Narrow alley").

"Do you have any questions?" Clarify any questions. "If you have no further questions, go to the start line with your bike and wait for my signal."

| **Station** | **Error category** | **Error class**  **(I/II)** | **Number of errors** |
| --- | --- | --- | --- |
| **1. Slalom** | Touching of cone | 3x |  |
|  | Omission of cone | 3x |  |
|  | Foot-to-floor contact | 3x |  |
|  | Cycling in a standing position | 1x |  |
| **2. Slow cycling**  Time: _________ (s) | Touching the sideline | 3x |  |
|  | Foot-to-floor contact | 3x |  |
|  | Cycling in a standing position | 1x |  |
|  | Time error (x < 10 sec) | 1x |  |
| **3a. Dismounting right** | Cycling in a standing position | 1x |  |
|  | Foot-to-floor contact outside of the hoop/ Touching the hoop with the foot/bicycle | 3x |  |
| **3b. Mounting right** | Foot-to-floor contact outside of the hoop/ Touching the hoop with the foot/bicycle | 3x |  |
| **3c. Dismounting left** | Cycling in a standing position | 1x |  |
|  | Foot-to-floor contact outside of the hoop/ Touching the hoop with the foot/bicycle | 3x |  |
| **3d. Mounting left** | Foot-to-floor contact outside of the hoop/ Touching the hoop with the foot/bicycle | 3x |  |
| **4. Narrow alley** | Foot-to-floor contact | 3x |  |
|  | Touching the sideline | 3x |  |
|  | Cycling in a standing position | 1x |  |
| **5. Turning to the left** | No hand signal/ Hand signal too late | 1x |  |
|  | No shoulder check | 1x |  |
|  | No/incorrect naming of the number | 1x |  |
|  | Touching the sideline | 3x |  |
|  | Foot-to-floor contact | 3x |  |
|  | Cycling in a standing position | 1x |  |
| **6.** **Cycling a figure eight** | Foot-to-floor contact | 3x |  |
|  | Touching the sideline | 3x |  |
|  | No eight on the first attempt | 1x |  |
|  | Cycling in a standing position | 1x |  |

Notes:

6. Analysis

**General information**

- Measuring point:
  - The measuring point on the bike is always the contact point between the tire and the ground and not the start of the front wheel (i.e. not like a light barrier measurement; e.g. timing the “Slow speed” station or error category “Crossing the line”).
- Error classes:
  - The respective error categories are assigned to 2 error classes:
    - Error class I errors can only be committed once per station (1x).
    - Error class II errors can be committed up to three times per station (3x).
- Follow-up errors:
  - Certain error categories (= error class II) can be committed multiple times (maximum 3 times) per station. If the participant regains control of the bicycle after committing the first error in a certain category (e.g. “Touching the sideline”) and then makes the same error again, the respective error category can be assigned again. If additional errors of other error categories are committed in the course of this loss of control (e.g. additional “Foot-to-floor contact”), these errors are also assigned as long as they do not occur simultaneously (see multi-errors). If the participant was unable to regain control of the bicycle after committing the first error and makes the same error again in the subsequent process, the respective error category is not assigned again in the sense of a follow-up error.
  - If the test participant slowly pushes forward on the bicycle with their feet instead of using the pedals, the error “Foot-to-floor contact” is assigned up to 3 times within the respective station and is not counted as a follow-up error.
- Multi-errors:
  - If several errors of different categories are committed at the same time, only one error is assigned in total (e.g. “Touching the sideline” with simultaneous “Foot-to-floor contact”).
- Maximum error count:
  - The maximum error count per station is the sum of all possible errors of the individual error categories of the respective station (e.g. station 4: 3x “Foot-to-floor contact” + 3x “Touching the sideline” + 1x “Cycling in a standing position” = 7 errors).
  - Loss of control:

*The participant loses control of the bicycle while cycling and does not regain control through use of regulating support measures (braking, counter-steering, simple foot-to-floor-contact).*

*EITHER*

1. *The participant then unintentionally comes to rest on the ground or another lower level (e.g. accidentally slips of pedals or saddle). If this (= a fall) occurs, the maximum error count for the respective station is assigned.*

*OR*

2. *If the participant deliberately gets off the bike within a station due to being overwhelmed or insecure and then skips it or restarts it completely, the maximum number of faults for the respective station is applied. 🡪 skipping or restarting completely shows us that the participant is not able to complete or to do the task at all*

- Touching the line constantly during the whole station: one error is assigned because keeping balance is proven

**Station-specific rules**

1. **Station: Slalom:**
   - If the participant pushes through the station with their feet instead of using the pedals, the errors ““Foot-to-floor contact” and “Omission of cone” are assigned up to 3 times each, depending on the number of cones passed.
   - If the participant commits an error (e.g. “Foot-to-floor contact”, fall after the last cone) in the process of exiting the slalom (i.e. immediately after the station), these will still be assigned.
2. **Station: Slow cycling:**
   - If the participant enters and/or exits the slow cycling corridor too late or too early the time measurement starts and/or stops when the sideline is crossed.
3. **Station: Dis-/mounting:**
   - Foot-to-floor contact between stations 2 and 3 is considered an error in station 3a (protocol).
   - Foot-to-floor contacts between stations 3b and 3c are split in half. Foot-to-floor contacts in the first half of the distance between stations 3b and 3c are assigned to station 3b. After that, further Foot-to-floor contacts are assigned to station 3c.
   - If the participant starts station 3a on the wrong side (= left), no error is assigned if the participant then performs the exercise correctly and corrects him/herself and completes station 3c on the opposite side (= right). If the participant does not correct him/herself and dismounts to the same side again, this is counted as omitting the second station (3c + 3d) and the maximum error count is assigned (4+3 = 7)
   - Immediately after station 3d, up to three foot-to-floor contacts can be assigned during the process of cycling off.
4. **Station: Figure 8**

- Touching the line constantly with the rear tire during the whole station: one error is assigned because keeping balance is proven.
