# Supplementary material for: Outdoor cycling competence and balance control in older adults – cross-sectional analysis of the BiPerAge trial
Source: BMC Geriatr. 2026 Oct 19;26:1306. doi: 10.1186/s12877-026-08491-7 (PMC13682123; doi:10.1186/s12877-026-08491-7)
Supplement: Supplementary file 2 [file 12877_2026_8491_MOESM2_ESM.docx]

**Perturbation test**

| Time [s] | Velocity [mm/s] | Angle [degree] | Direction |
| --- | --- | --- | --- |
|  |  |  |  |
| 180 | 20 | 1 | l |
| 195 | 20 | 1 | r |
| 210 | 20 | 1 | b |
| 220 | 10 | 1 | f |
| 230 | 20 | 1 | l |
| 240 | 30 | 2 | b |
| 255 | 30 | 2 | r |
| 270 | 20 | 2 | f |
| 280 | 30 | 2 | b |
| 290 | 30 | 2 | r |
| 300 | 200 | 2 | l |
| 315 | 200 | 2 | r |
| 330 | 200 | 2 | b |
| 340 | 200 | 1 | f |
| 355 | 250 | 3 | l |
| 370 | 250 | 3 | b |
| 385 | 250 | 3 | r |
| 400 | 275 | 4 | b |
| 410 | 250 | 2 | f |
| 420 | 275 | 4 | r |
| 435 | 275 | 4 | l |
| 455 | 300 | 5 | r |
| 470 | 300 | 5 | b |
| 485 | 300 | 3 | f |
| 500 | 300 | 5 | l |
| 515 | 350 | 6 | b |
| 530 | 350 | 6 | l |
| 545 | 350 | 6 | r |
| 560 | 350 | 4 | f |
| 575 | 400 | 7 | l |
| 590 | 400 | 7 | r |
| 605 | 400 | 7 | b |
| 620 | 400 | 5 | f |
| 635 | 450 | 8 | l |
| 650 | 450 | 8 | r |
| 665 | 450 | 8 | b |
| 680 | 475 | 9 | r |
| 695 | 475 | 9 | f |
| 710 | 450 | 6 | f |
| 725 | 475 | 9 | l |
| 740 | 500 | 10 | l |
| 755 | 500 | 10 | b |
| 775 | 500 | 10 | r |

*r* right; *l* left; *f* forward; *b* backward
